# A novel BMX variant promotes tumor cell growth and migration in lung adenocarcinoma

**DOI:** 10.18632/oncotarget.16796

**Published:** 2017-04-03

**Authors:** Ye Wang, Jufeng Xia, Zhaoyuan Fang, Fei Li, Duo Li, Zuoyun Wang, Yan Feng, Jian Zhang, Haiquan Chen, Hongbin Ji, Hongyan Liu

**Affiliations:** ^1^ CAS Key Laboratory of Systems Biology, Institute of Biochemistry and Cell Biology, Shanghai Institutes for Biological Sciences, Chinese Academy of Science, Shanghai, 200031, China; ^2^ CAS Center for Excellence in Molecular Cell Science, Institute of Biochemistry and Cell Biology, Shanghai Institutes for Biological Sciences, Chinese Academy of Science, Shanghai, 200031, China; ^3^ Innovation Center for Cell Signaling Network, Institute of Biochemistry and Cell Biology, Shanghai Institutes for Biological Sciences, Chinese Academy of Science, Shanghai, 200031, China; ^4^ Institute of Basic Medicine, Shandong Academy of Medical Sciences, Jinan, 250062, Shandong, China; ^5^ School of Life Science and Technology, Shanghai Tech University, Shanghai, 200120, China; ^6^ Department of Thoracic Surgery, Fudan University Shanghai Cancer Center, Shanghai, 200032, China; ^7^ Department of Oncology, Shanghai Medical College, Fudan University, Shanghai, 200032, China

**Keywords:** lung adenocarcinomas, BMXΔN, cell proliferation, migration, skipping variant

## Abstract

The non-receptor tyrosine kinase BMX has been reported in several solid tumors. However, the alternative splicing of *BMX* and its clinical relevance in lung cancer remain to be elucidated. Exon1.0 array was used to identify a novel alternative splicing of *BMX, BMXΔN*, which was confirmed by rapid amplification of cDNA ends and reverse transcription-polymerase chain reaction. *BMXΔN*, resulting from exon skipping with excluding exon 1 to exon 8 of *BMX* gene, was found in 12% human lung adenocarcinoma specimens. *BMXΔN* is not found in paired pathologically normal lungs and positively correlated with *EGFR* mutation in lung adenocarcinomas. Moreover, BMXΔN increases cell proliferation, neoplastic transformation, and migratory property of human non-small cell lung cancer cells. The function of BMXΔN in lung cancer might be presumably due to enhanced ERK signaling.

## INTRODUCTION

Alternatively spliced proteins are particularly relevant in oncology since they have been linked to cancer progression and drug resistance [1, 2]. They may provide selective drug targets, or serve as a marker set for cancer diagnosis as well. For example, the tumor suppressor gene *p53* is subject to alternative splicing and p53 splice variants are frequently expressed in primary ovarian cancers [3]. The *p53δ*, encoding a C-terminally truncated protein, was demonstrated to be associated with impaired response to primary platinum-based chemotherapy and might serve as an adverse prognostic marker for recurrence free and overall survival in ovarian cancers [3]. A splicing variant of Merlin, ^Δ2-4^Merlin, promotes tumor metastasis by interfering with the tumor suppression role of wild type Merlin [4]. There is substantial evidence that primary metabolism is altered in cancer cells, and the pyruvate kinase M1 and M2 splicing isoforms control the balance between aerobic and anaerobic glycolysis during tumor progression [5, 6]. These observations emphasized the importance of investigation of alternative splicing genes in cancer for improving targeted therapy.

Affymetrix Exon1.0 array detects gene expression at single exon level. This facilitates the identification of alternative splicing isoform of certain genes as well as the gene fusions. Our previous study has analyzed the Exon1.0 array from 76 Chinese lung adenocarcinomas and identified CCDC6-RET fusion as novel oncogenic driver [7]. Certainly, except for the gene fusion, detection of alternative splicing is another outcome of this dataset.

*BMX* (bone marrow tyrosine kinase gene in chromosome X), which encodes a non-receptor tyrosine kinase belonging to *BTK* (Bruton's tyrosine kinase) family. BMX has been shown to play a pivotal role in the regulation of various cellular processes including proliferation, differentiation, transformation, apoptosis, and cell motility. Previous study described BMX as a direct substrate for caspases and the resulting truncated molecule contains an intact SH2 domain and kinase domain which has an enhanced kinase activity [8]. BMX acts upstream of RhoA and activates RhoA by releasing GDI from the RhoA-GDI complex through the interaction between the PH domain of BMX and RhoA [9]. BMX directly associates with Pak1 via its N-terminal pleckstrin homology domain and also phosphorylates Pak1 on tyrosine residues [10]. Study has also shown that BMX interacts with p53 in response to DNA damage and that such interaction leads to bidirectional inhibition of the activities of both proteins in LNCaP human prostate carcinoma cells [11]. Studies also illustrated some of the upstream activator for BMX. For example, BMX activity is modulated by FAK through an interaction between the PH domain of BMX and the FERM domain of FAK and the activation of BMX by FAK promotes cell migration [12]. In addition, BMX can be induced by growth factors, cytokines [13], the extracellular matrix, and possibly by hormones [14]. More importantly, BMX mediates various signaling pathways including STAT signaling pathway [15, 16], PI-3K signaling pathways [17–19], and GPCR signaling pathway [20].

BMX expression is altered in a number of different cancers, including those of the breast and prostate [10, 21–23], suggesting BMX may play roles in cancers. For example, BMX expression level is up-regulated in hormone-resistant prostate cancer and positively correlated with tyrosine phosphorylation of AR conditions. Overexpression of BMX in androgen-sensitive LNCaP cells promotes tumor growth while knocking down BMX expression in hormone-insensitive prostate cancer cells inhibits tumor growth under androgen-depleted conditions [24].

Here we describe the discovery of a novel spliced variant of *BMX*, designated as *BMXΔN*, which results from the skipping exon 1 to exon 8 in *BMX* gene. *BMXΔN* is strongly associated with *EGFR* mutation in clinical samples. Moreover, this isoform promotes lung cancer cell growth, migration, and neoplastic transformation.

## RESULTS

### Identification of a novel *BMX* skipping isoform in lung adenocarcinoma

**Figure 1:**
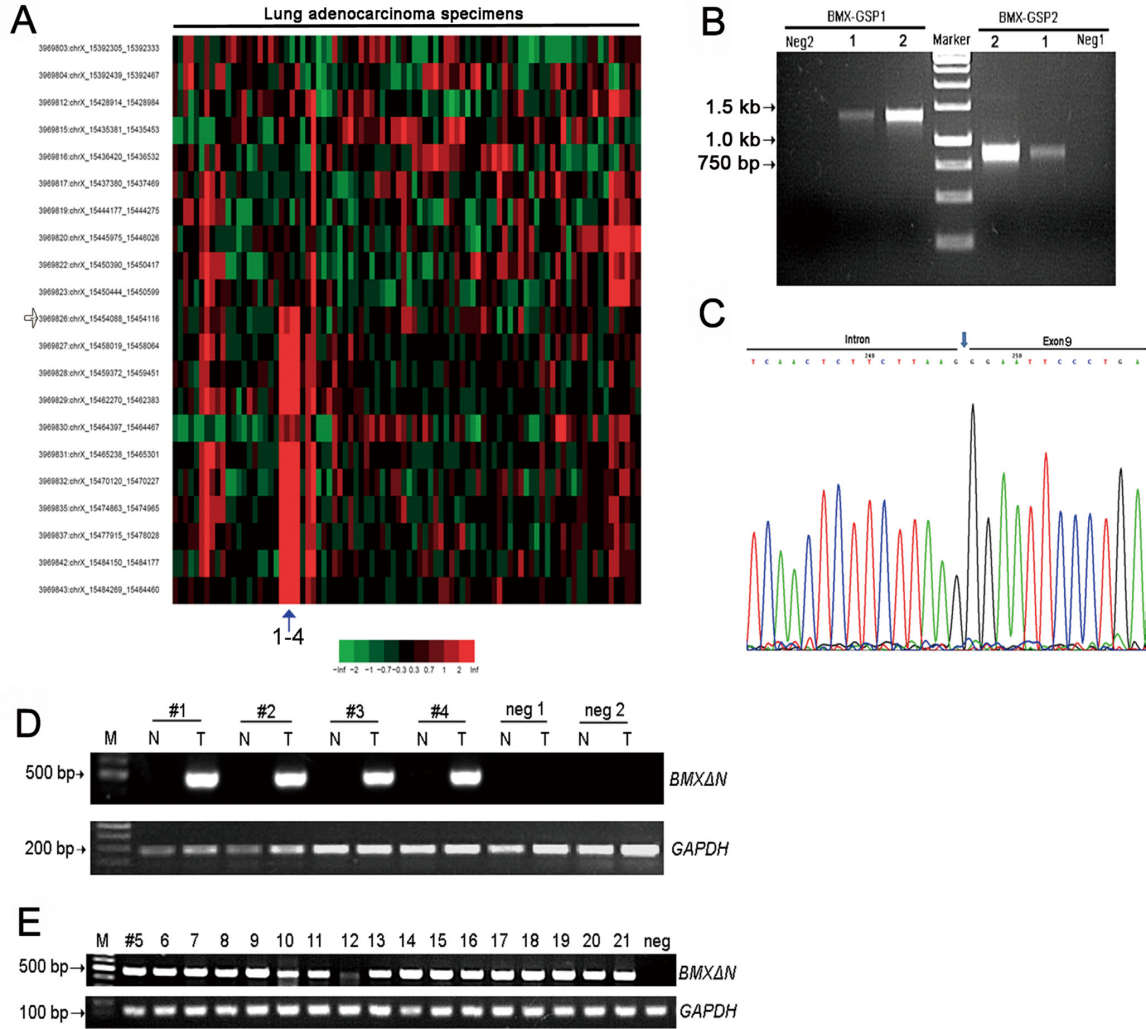
Identification of a novel BMX skipping isoform in human lung adenocarcinomas (**A**) Exon array analyses of 78 lung adenocarcinoma samples and 10 paired non-cancerous lung samples have identified *BMX* abnormal splicing in lung adenocarcinoma sample 1 to 4. The potential break point was indicated by the arrow. (**B**) 5′ RACE analyses of the lung adenocarcinoma sample1 and sample 2 using two specific *BMX* primers showed the sharp PCR bands (> 750 bp and > 1300 bp), which is different from the predicted wild type *BMX* band (about 695 bp and 1177 bp from primer location to breakpoint). (**C**) Sequencing result confirmed the *BMX* abnormal splicing in lung adenocarcinoma sample 1and sample 2. The sequencing result showed the detailed N-terminal sequence of *BMX* lacking exon 1 to exon 8 but retaining part of intron 8. (**D**) The representative data showed that *BMXΔN* existed in lung adenocarcinomas but not in paired non-cancerous lung samples and control samples (negative 1 and negative 2). (**E**) Specific RT-PCR showed the detection of *BMXΔN* in another 17 lung adenocarcinomas identified from 174 lung adenocarcinomas.

Through bioinformatics analyses of Exon1.0 array data from Chinese lung adenocarcinoma and 5′ RACE, we identified a novel *BMX* skipping variant (Figure 1A, 1B). We called this novel *BMX* isoform, *BMXΔN*, which lacked the N-terminal sequence from exon 1 to exon 8 (Figure 1C). We further found that *BMXΔN* was absent in all the 14 paired non-cancerous lung tissues. Representative reverse transcription-PCR analysis showed that *BMXΔN* was detectable in lung adenocarcinomas but not in paired non-cancerous lung samples (Figure 1D). Then, we expanded the study of *BMXΔN* in a cohort with 174 adenocarcinoma samples and identified a total of 21 lung adenocarcinomas harboring this isoform (12%, 21/174) (Figure 1E).

### Detection of *BMXΔN* translation start codon

**Figure 2:**
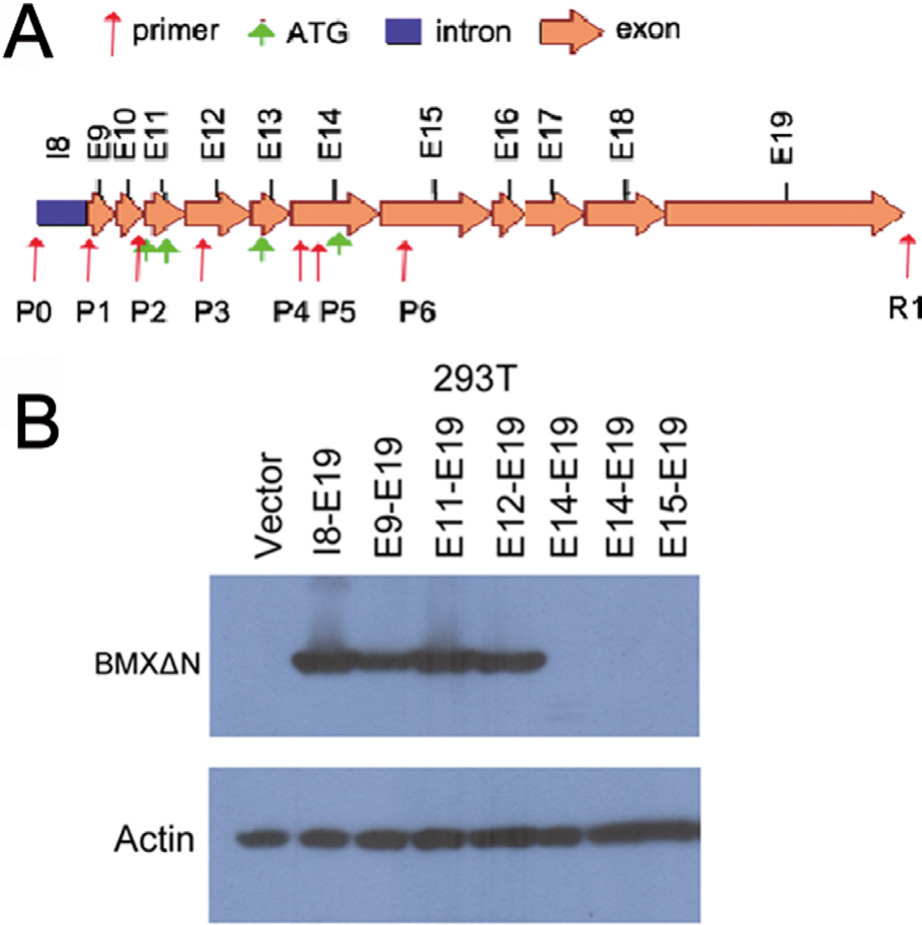
Detection of BMXΔN translation start codon (**A**) Schematic representation of the predicted start codon of *BMXΔN*. The positions of exons are indicated by arrows. The positions of 7 forward primers (P0 to P6) and 1 reverse primer (R1) for PCR are also indicated below the corresponding intron or exons. (**B**) Immunoblot analysis of BMXΔN and actin in HEK-293T cells. Cell lysates of HEK-293T cells transfected with the indicated cDNAs in expression vectors were analyzed.

The sequence of the *BMXΔN* gene contains four putative start codons (ATG_1_-ATG_4_). We detected at which ATG codon BMXΔN translation initiates. We constructed a series of plasmids with different ATGs and then transfected the plasmid into HEK-293T cells (Figure 2A). Western blot analysis of total protein from HEK-293T cells showed that BMXΔN was translated from plasmid carrying ATG_3_ (Figure 2B), indicating that the ATG located in exon 13 is the start codon for *BMXΔN*.

### The relationship between *BMXΔN* expression and *EGFR* mutation

We further analyzed the relationship between *BMXΔN* expression and clinicopathological features in human lung adenocarcinomas (Tables 1 and 2). *BMXΔN* expression was not significantly correlated with age, gender, pathological stage (Table 2) and metastasis (Table 1). However, we found that *BMXΔN* was tightly associated with *EGFR* mutation (*p* = 0.002). Indeed, 20 out of 21 samples harbor *EGFR* mutation (Table 2).

**Table 1:** Correlation of BMXΔN expression with patients’ clinicopathological variables in 146 cases of adenocarcinomas

Characteristics	Number of cases	BMXΔN expression		*P*-value
		Yes	No	
Age (years)				
> = 50	117	12(10.3%)	105(89.7%)	
< 50	29	7(24.1%)	22(75.9%)	0.063
Gender				
Male	40	6(15%)	34(85%)	
Female	106	13(12.3%)	93(87.7%)	0.661
T Classification				
T1–2	131	18(13.7%)	113(86.3%)	
T3–4	15	1(6.7%)	14(93.3%)	0.441
LN metastasis				
Negative	90	12(13.3%)	78(86.7%)	
Positive	56	7(12.5%)	49(87.5%)	0.884
Clinical Stage				
I–II	98	12(12.2%)	86(87.8%)	
III–IV	48	7(14.6%)	41(85.4%)	0.693
*EGFR* mutation				
Wild type	44	0(0%)	44(100%)	
Mutation	102	19(18.6%)	83(81.4%)	0.002

Only patients with detailed pathological data were compared in statistical analysis.

**Table 2:** The clinical relevance of BMXΔN expression

Sample	Gender	Age (years)	Pathological stage	EGFR mutation
1	M	60	IIIb	L858R
2	M	63	Ia	L858R
3	F	59	IIa	L858R
4	F	54	Ib	L858R
5	M	46	Ia	746ELREA deletion
6	F	48	Ia	746ELREA deletion
7	F	68	IIIa	L858R
8	M	57	IIIa	L858R
9	M	46	IIa	746ELREATS = > V 753P = > Q
10	F	46	IIIa	L858R
11	F	58	Ib	747LREA deletion
12	M	46	IV	L858R
13	F	76	Ia	746ELREA deletion
14	F	51	Ib	746ELREA deletion
15	M	76	Ia	L858R
16	F	52	Ia	L858R
17	F	39	IIIa	746ELREA deletion
18	F	47	Ia	L858R
19	N/A	N/A	Ia	No (HER2 mutation)
20	F	55	Ia	L858R
21	F	48	IIIa	746ELREA deletion
*P*-value	0.661	0.063	0.693	0.002**

Abbreviations: F, female; M, male; N/A, not available.

### Low expression of *BMX* in lung adenocarcinomas

**Figure 3:**
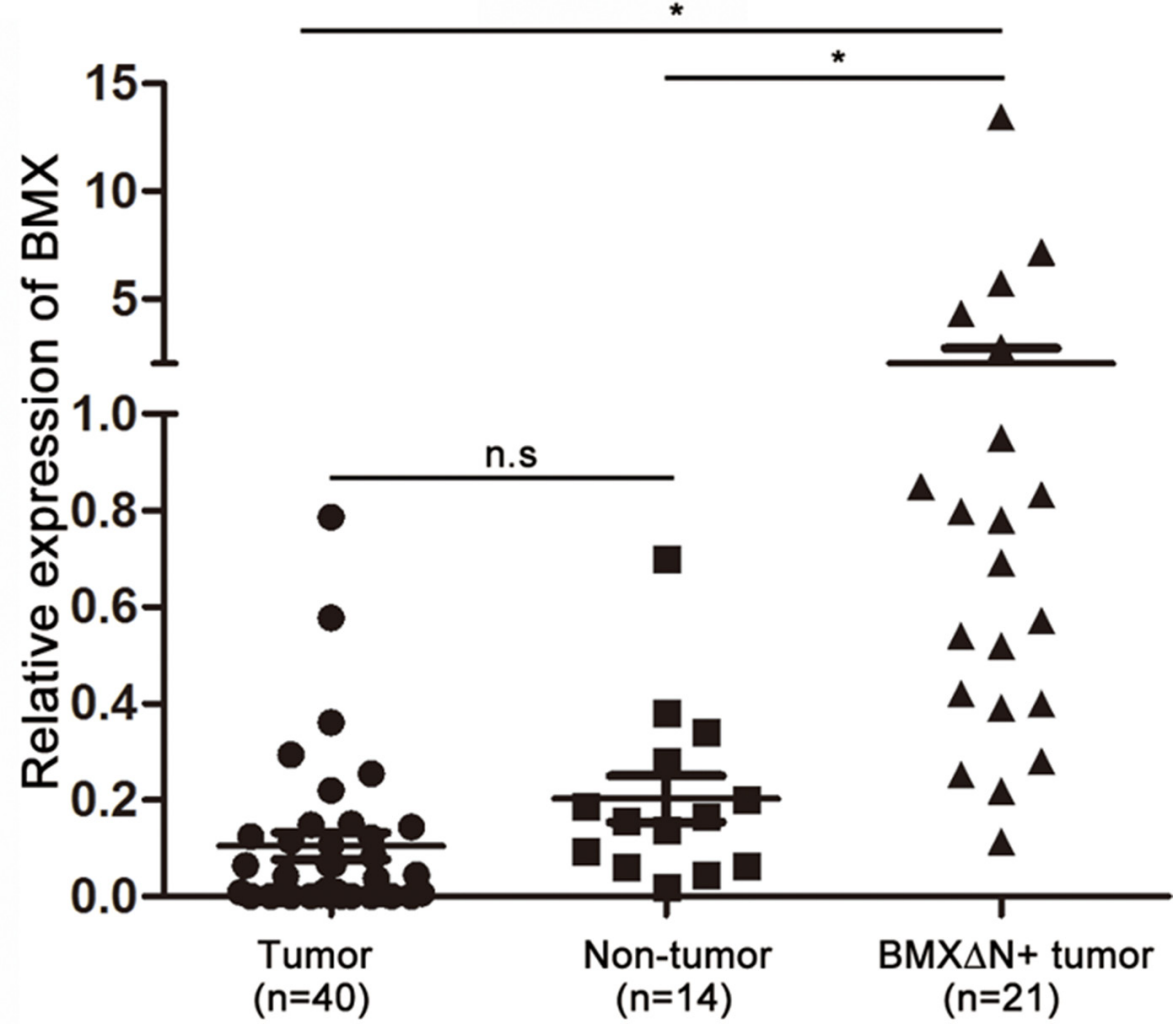
Expression of the *BMX* gene in lung adenocarcinomas Detection of wild type *BMX* and *BMXΔN* in non-tumour adjacent tissues, lung adenocarcinomas without *BMXΔN* and with *BMXΔN* tissues by primers designed from the *BMX* exon 16 and 17. n.s, not significant; *P < 0.05, based on the student's *t*-test.

On the basis of previous studies showing three transcript variants of *BMX*, a pair of primers (F8/R12) was designed encompassing exons 8 to 12 of the *BMX* open reading frame for detection of wild type *BMX* and other two variants. Another pair of primers (F16/R17) was also designed encompassing exons 16 and 17 of the *BMX* open reading frame to detect all *BMX* isoforms including *BMXΔN*. Using F16/R17 primers to observe the levels of *BMX* mRNA in lung adenocarcinomas and adjacent non-tumour specimens by quantitative PCR, we found that there were no significant differences in expression. However, the transcript of *BMX* was different between *BMXΔN* positive lung adenocarcinomas and adjacent non-tumour specimens (Figure 3). Interestingly, when we use F8/R12 primers to detect the levels of *BMX* mRNA in *BMXΔN* positive lung adenocarcinomas, we found very low expression of *BMX* or even no expression of *BMX* in these lung adenocarcinomas (data not shown). These findings indicate that *BMXΔN* is the dominant isoform in these specimens. Because wild type BMX functions as an oncogene in prostate cancer [24], we decided to explore the role of BMXΔN in our study of lung carcinogenesis.

### Effect of BMXΔN on NSCLC cell growth

**Figure 4:**
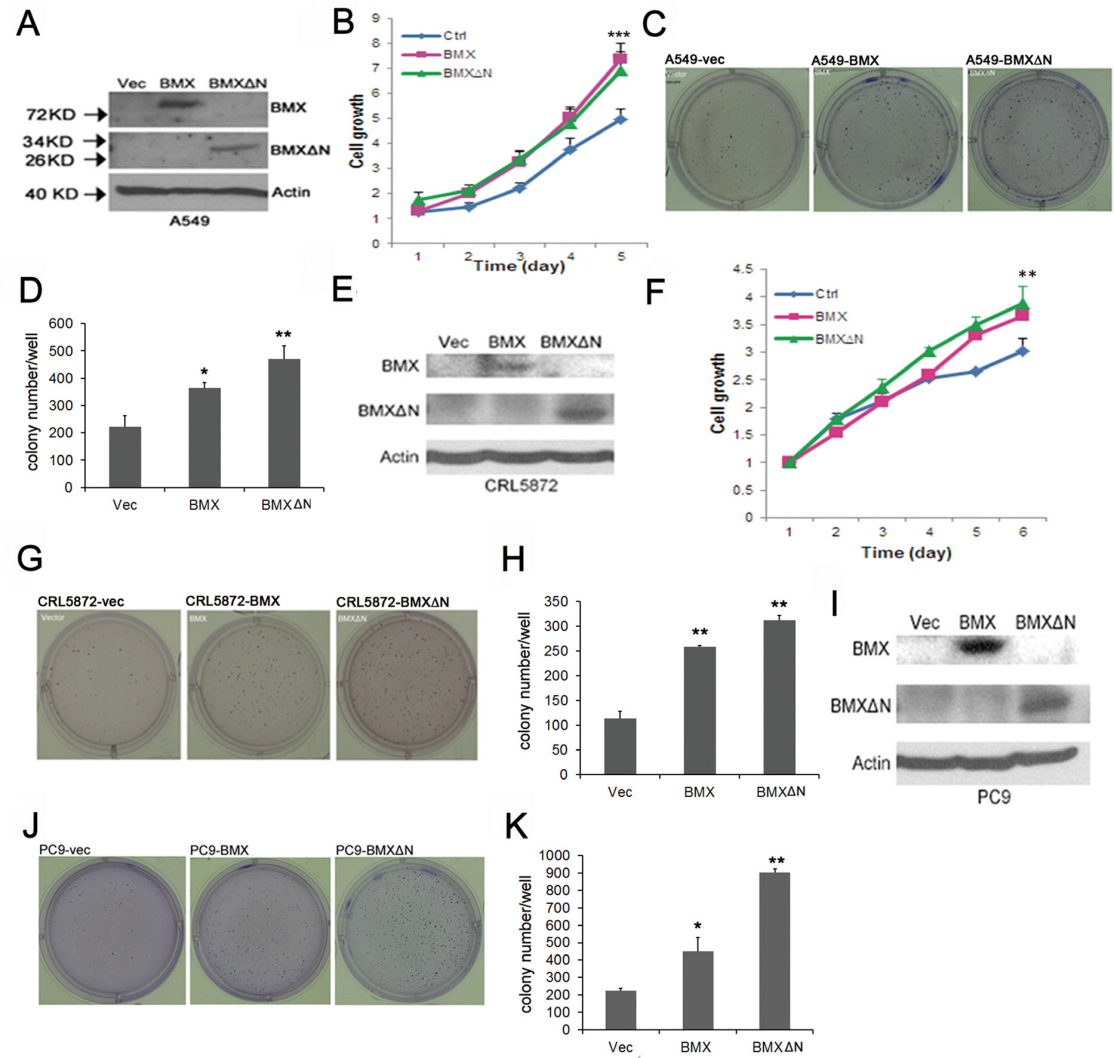
BMXΔN expression promotes cell growth and transformation (**A, E** and **I**) Immunoblot analyses of BMX and BMXΔN expression in A549, CRL-5872 and PC9 cells after enforced expression of BMX or BMXΔN. (**B** and **F**) MTT assay of cell viability in A549 and CRL-5872 cells after enforced expression of BMX or BMXΔN. Statistical analysis was performed using Student's *t* test (*P* value is **P < 0.01, ***P < 0.001). (**C, D, G, H, J** and **K**) Soft Agar assay of A549, CRL-5872 and PC9 cells after enforced expression of BMX or BMXΔN. Representative images of colony formation are shown in (C, G, J) and quantitation of colonies is shown in (D, H, K). Statistical analysis was performed using Student's t test (*P* value is *P < 0.05, **P < 0.01).

To investigate the role of BMXΔN in cell growth, we performed cell proliferation assay on A549, CRL-5872, and PC9 cells (Figure 4). Expression of BMXΔN efficiently promoted cell growth in A549 (Figure 4B). The wide type BMX elevated cell growth as well. To examine whether BMXΔN is involved in cell transformation, we performed soft agar colony formation assays on these cells. Figure 4C shows that more colonies were formed in BMXΔN expressing cells compared with control. Similar growth promotion effect and transformation activity were also observed in CRL-5872 cells (Figure 4F–4H). Although BMXΔN did not increase cell proliferation in PC9 cells (Supplementary Figure 1), it induced colony formation in soft agar (Figure 4J, 4K). These data suggested that BMXΔN promoted lung cancer cell growth *in vitro*.

### BMXΔN facilitates tumor cell migration and enables cell transformation

**Figure 5:**
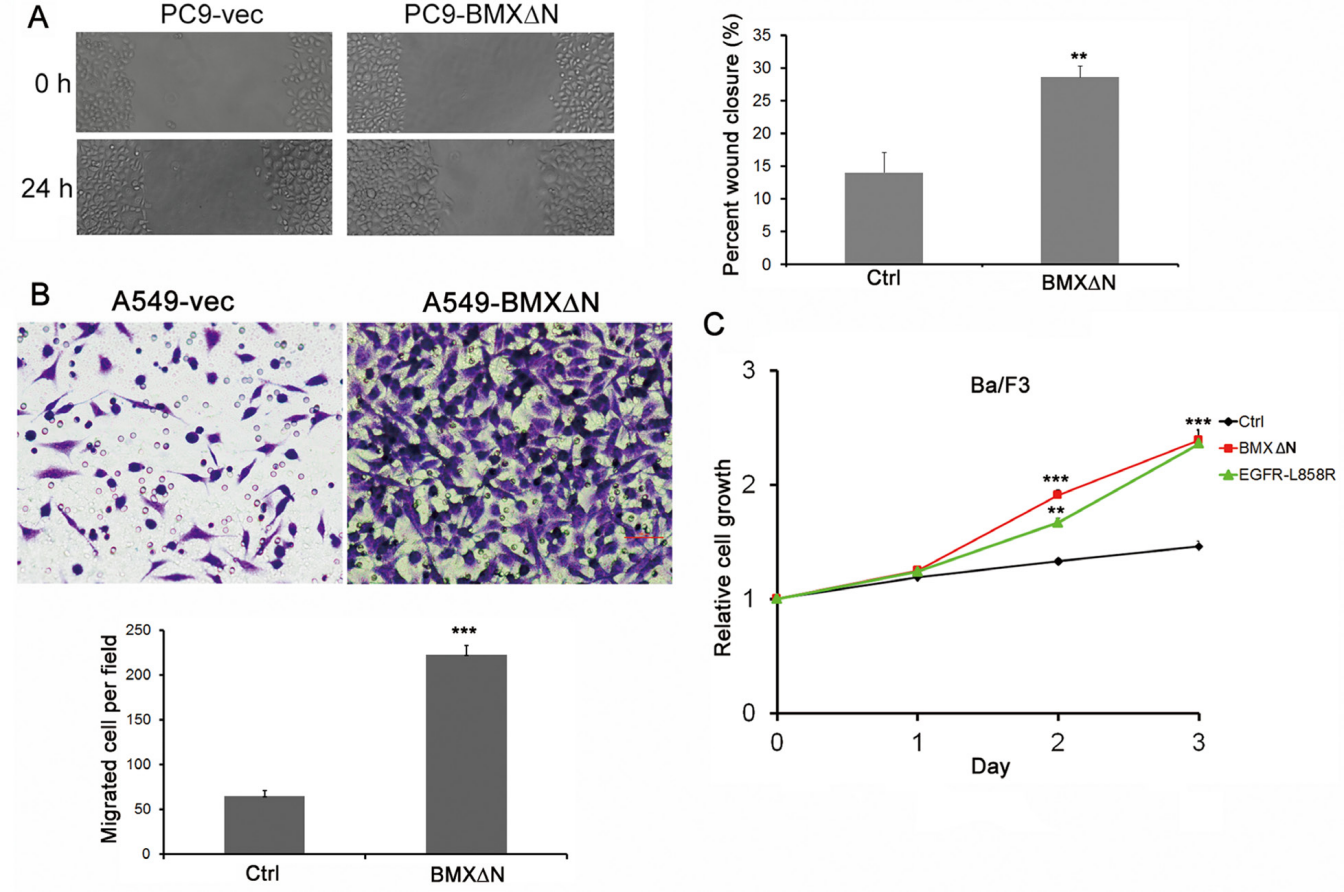
BMXΔN promotes cell migration and Ba/F3 transformation (**A**) Wound healing assay of PC9 cells with or without BMXΔN expression. Representative images of cell migration are shown at 0 h and 24 h. (**B**) BMXΔN promoted A549 cell migration in transwell assay. Statistics analyses were shown below. (**C**) The effect of BMXΔN and EGFR-L858R on Ba/F3 cell transformation was examined at time indicated. Statistical analysis was performed using Student's t test (*P* value is **P < 0.01, ***P < 0.001).

We further studied the role of BMXΔN in lung cancer cell migration (Figure 5A, 5B). The wound-healing assay showed BMXΔN transfected PC9 cells obtained quicker closure of the scratched “wound” compared with control cells (Figure 5A). Migration was also examined using transwell assays where the cells were incubated in serum-free DMEM medium in the upper compartment and allowed to migrate towards the lower compartment containing 15% FBS. The result showed that enforced BMXΔN expression greatly increased the migration ability of A549 cells (Figure 5B). To evaluate the transformation capacity of BMXΔN, we introduced BMXΔN and mutant EGFR into Ba/F3 cells. *BMXΔN*-transfected Ba/F3 cells showed accelerated growth rate compared with mock transfectants, whereas no difference in growth was observed between the *BMXΔN*- and *EGFR L858R*-transfected cells. These results indicate that BMXΔN was capable of transforming Ba/F3 cells *in vitro* (Figure 5C). The above findings demonstrated an important role of BMXΔN in lung cell carcinogenesis.

### BMXΔN activated ERK in lung cancer cells

**Figure 6:**
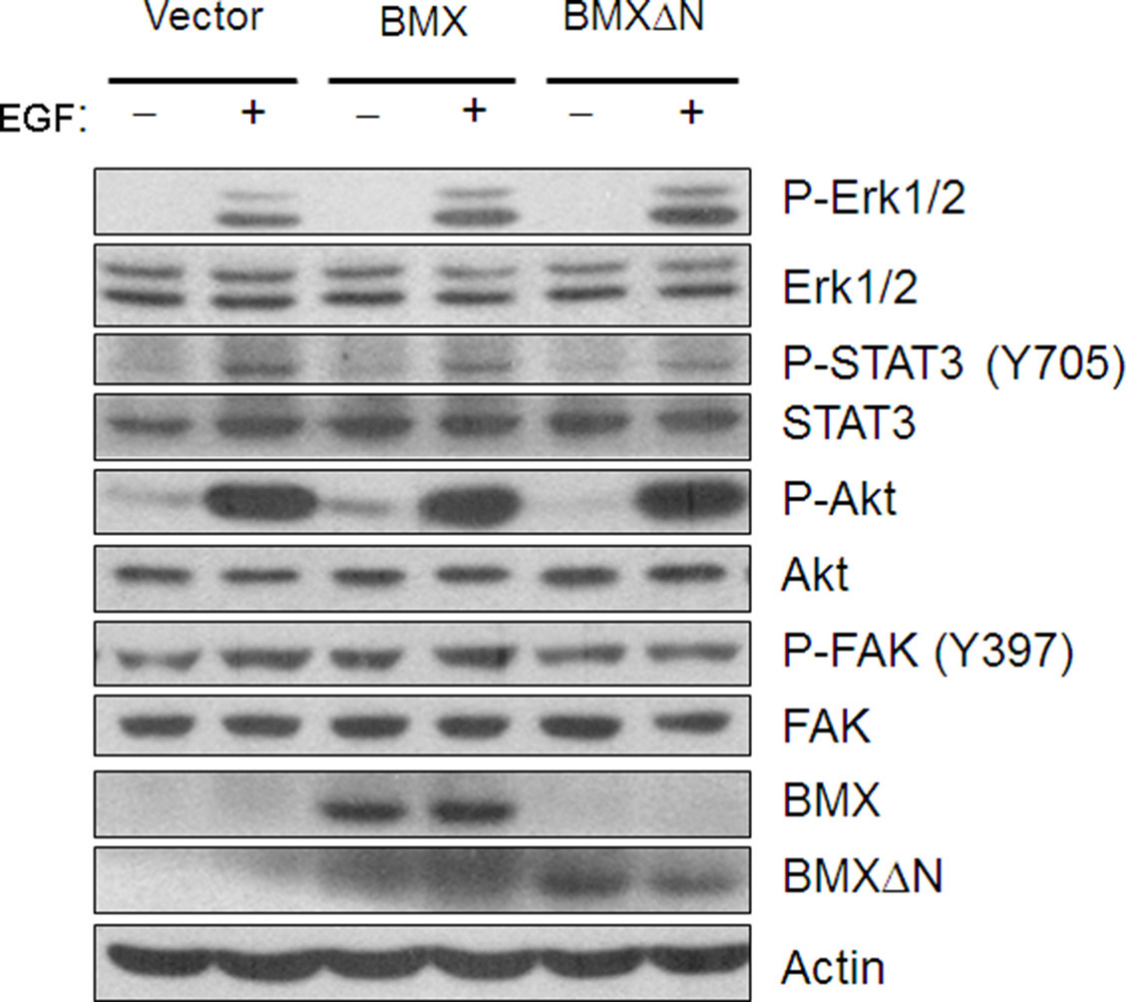
BMXΔN expression activates ERK in lung cancer cells Immunoblot analysis is performed to detect BMX/BMXΔN protein expression and phosphorylation of ERK, STAT3, AKT and FAK signaling pathway in A549 cells with or without BMX/BMXΔN expression. Cells were treated with EGF (50ng/ml) for two hours before extraction of total cellular protein.

Previous studies have shown that BMX expression could activate several signaling pathways, including PI3-AKT pathway and STAT pathway. We tested if these pathways are also involved in BMXΔN function in lung cancer. We detected the phosphorylation of STAT3, ERK, AKT and FAK in lung cancer cells expressing either wild type BMX or BMXΔN and control cells followed by EGF stimulation. As shown in Figure 6, the much higher level of expression of phosphorylated ERK could be detected in BMXΔN transfected cells, suggesting MAPK pathway might contribute to the role of BMXΔN in lung cancer. Our data indicated that other tested pathways were not affected by BMXΔN expression in A549 cells.

## DISCUSSION

The discovery of alternative splicing variants in cancers has been paid much attention recently and their detection potentially increases with the use of innovative approaches. We here identified a *BMX* skipping isoform through the analyses of Exon1.0 array profiling of human lung adenocarcinoma samples in combination with RACE method. *BMXΔN* lacks the N-terminal sequence from exon 1 to exon 8. *BMXΔN* only expresses in lung cancer but not in paired non-cancerous tissues. Through screening a large collection of NSCLC patient samples, we identified a total of 21 lung adenocarcinomas expressing *BMXΔN* (12%, 21/174). Interestingly, *BMXΔN* is strongly associated with *EGFR* mutation in our study. However, the clinical relevance is not known yet.

A few studies have correlated BMX function with tumor growth, metastasis or poor prognosis in cancer. Overexpression of BMX in androgen-sensitive LNCaP cells promotes tumor growth while knocking down BMX expression in hormone-insensitive prostate cancer cells inhibits tumor growth under androgen-depleted conditions [21]. BMX is up-regulated in bladder cancer and predicts poor prognosis in patients with cystectomy [25]. A study has shown that BMX could maintain self-renewal and tumorigenic potential of glioblastoma stem cells by activating STAT3 [26]. Here, we present evidence that *BMX* was not up-regulated in lung adenocarcinomas. It might not contribute to lung carcinogenesis despite knowing it could promote cell proliferation of NSCLC. Importantly, our results reveal a previously unknown splicing skipping form of *BMX*. The studies suggested that BMXΔN might play roles in lung tumorigenicity, with expression of BMXΔN promoting cell growth, cell migration, and cell transformation. Future studies are necessary to clarify the mechanism by which BMXΔN activates ERK1/2.

Collectively, this study discovered a novel *BMX* skipping with crucial function in lung cancer cells. Future studies into this novel *BMX* variant might provide a better understanding of lung tumorigenesis and clinical implication for therapeutics.

## MATERIALS AND METHODS

### Specimen collection

The study was approved by the ethics review board at Fudan University Shanghai Cancer Center, Shanghai, China. 174 cases of lung adenocarcinomas with paired pathological normal lungs were collected consecutively with written informed consents from all patients. Fresh surgical specimens were snap-frozen and stored in liquid nitrogen upon resection until use. The pathology of each tumor sample was determined by pathologists. All these specimens were with a minimum of 70% of tumor cellularity, and all patients did not receive neoadjuvant chemotherapy. The status of *EGFR* mutations and other drive mutations in these specimens was determined as previously described [27, 28]. The correlation of *BMXΔN* expression and patients’ clinical characteristics were illustrated in 146 lung adenocarcinoma samples, a subset of 174 cases, containing 19 *BMXΔN* positive samples (Table 1).

### Cell culture, DNA constructs and plasmid transfection

A549, CRL-5872 and PC9 cells were purchased from the ATCC. Cells were cultured in DMEM, supplemented with 8% Fetal Bovine Serum (FBS), 100 μg/ml streptomycin and 100 U/ml penicillin, at 37°C in 5% CO2 incubator. A lentiviral construct expressing wild type *BMX* or *BMXΔN* were generated by cloning a DNA fragment corresponding to *BMX* full length or *BMX* residues 384-675 (NP-001712.1) into the NheI and NotI sites of pCDH-CMV-copGFP vector (SBI). Viral particles were produced in HEK-293T cells co-transfected with pCDH constructs and packaging plasmids pCMV-VSVG/delta8.2 (System Biosciences) in DMEM media. The progeny viruses released from HEK-293T cells were filtered, collected and used to infect cells.

### Gene functional assays

For cell proliferation assay, cells were seeded in 96-well plates at a density of 3×10^3^ cells per well, and cell growth rate was assessed with the 3-(4, 5-dimethylthiazol-2-yl)-2, 5-diphenyltetrazolium bromide (MTT) kit (Roche Diagnostics). The MTT assays in each cell line repeated three times, respectively. For soft agar colony formation assay, 8×10^3^ cells were seeded in 6-well plates, and after three weeks of culture cell colonies were counted by crystal violet staining. The results are expressed as the mean ± SD of three independent experiments. For wound-healing assay, PC9 cells were cultured on a 12-well plate and maintained in DMEM. At 80% to 90% confluence, the cells were starved for 12 hours cultured in DMEM without FBS. A 10 μl pipette tip was used to create a linear scratch. The cells were then washed with PBS to remove floating cellular debris and fed for an additional 24 hours with full DMEM. Migration photos were captured immediately after scratching and at 24 hours after scratching by a digital camera. Cell migration was also assessed using 12-well transwell chambers (Corning Costar) with a pore size of 8 μm. A549 cells (1 × 10^5^) were seeded in serum-free medium in the upper chamber and incubated at 37°C for 24 h. Afterward, the cells remained in the upper chamber were carefully removed with a cotton swab, whereas the cells having traversed to reverse face of the membrane were fixed with 5% acetic acid and stained with 0.4% crystal violet. Three random fields were counted at x20 magnification. The results represent the average of samples from three independent experiments. Oncogenic transformation assay was performed in Ba/F3 cells. Ba/F3 cells were infected with lentivirus containing a control vector or a *BMXΔN*/*EGFR-L858R* plasmid. Infected cells were incubated with IL-3 (0.5 pg/mL) to support Ba/F3 marginal growth for approximately 72 hours. 3000 Ba/F3 cells per well were plated in quadruplicate in 96-well plates and cultivated for 3 days without IL3. Cell viability was measured daily. The experiments were repeated independently three times.

### RT-PCR and quantitative real-time PCR

Total RNA was extracted from tissues with TRIzol^®^ Reagent (Invitrogen) and reverse transcribed into cDNA using the Superscript III Kit (Invitrogen) according to the manufacturer's instruction. The cDNAs were used as templates in PCR with *BMXΔN* gene-specific primers (forward primer, 5′-AGGGTGGGATTT GATATTGCATGG-3′ and reverse primer, 5′-CCAGGGA CACAGAGTCGGGGA-3′). The human glyceraldehyde-3-phosphate dehydrogenase gene (forward primer, 5′-GCGACACCCACTCCTCCACCTTT-3′; and reverse primer, 5′-TGCTGTAGCCAAATTCGTTGTCATA-3′) was used as an internal control in PCR amplification. The amplification reactions were performed using AmpliTaq Gold DNA polymerase (Applied Biosystems). The PCR program for detection of *BMXΔN* and *GAPDH* is: 95°C 5 minutes; 95°C 15 seconds, 58°C 30 seconds, 72°C 30 seconds, 35 cycles for *BMXΔN* and 32 cycles for *GAPDH*; 72°C 7 minute.

Real-time PCR was performed on an Applied Biosystems 7900HT cycler using SYBR Green Master Mix (SA Biosciences). The primers that were used for amplification of wild type *BMX* (NM_203281.2) and another two variants (NM_001721.6, NM_001320866.1) as follows: 5′-CAGTAACCAAAAAGAAAGAAATG-3′ and 5′-TGTGTTGATGATAATGAATAAGC-3′. The primers that were used for amplification of both wild type *BMX* and *BMXΔN* as follows: 5′-CTGCTCGTAAC TGCTTGGTGG-3′ and 5′-CTGACTTGCTGCTGTATT TGA-3′. The primers for housekeeping gene *GAPDH* were 5′- GCGACACCCACTCCTCCACCTTT-3′ (forward) and 5′- TGCTGTAGCCAAATTCGTTGTCATA-3′ (reverse); the primers for *ACTIN* were 5′-CTTAGTTGCGTT ACACCCTTTCT-3′ (forward) and 5′-TGCTGTCA CCTTCACCGTTC-3′ (reverse). The following conditions were used for PCR: i) initial denaturation step at 95°C for 8 min; and ii) 40 cycles at 95°C for 15 sec and 58°C for 45 sec. Relative quantity of *BMX* expression was calculated by the 2^−ΔΔCt^ method standardized to *GAPDH* or *ACTIN* expression in corresponding tissues.

### Exon array analysis and 5′ RACE

Exon array analysis and 5′ RACE were performed as previously described [7]. Briefly, Affymetrix Human Exon 1.0 microarray was used and the Robust Multichip Average method was applied to perform background correction, normalization and exon-level probe set summarization. 5′ RACE-PCR was performed using SMARTer^TM^ RACE cDNA Amplification Kit from Clontech Laboratories Inc (Mountain View, CA) according to the manufacturer's instructions. In brief, 1 μg RNA extracted from lung cancer patient tissues was reverse transcribed using primers 5′-RACE CDS primer A and SMARTer II A Oligonucleotide supplied by SMARTer^TM^ RACE cDNA Amplification Kit. PCR was performed with *BMX* gene specific primer 5′-GGTTCAAGTCCTTTTC CGTGACTCCTCA-3′/5′-CCCGAAGTGGTTCAATGGA AGACAGGA-3′ in conjunction with RACE universal primer A mix (UPM): 5′-CTAATACGACTCACTATA GGGCAAGCAGTGGTATCAACGCAGAGT-3′ and 5′-CT AATACGACTCACTATAGGGC-3′. PCR products were purified for direct sequencing as well as cloning into pGEM-T vector (Promega, Madison, WI) for sequencing.

### Western blot

Western blot analyses were performed according to the standard protocol. The following antibodies were used: pEGFR (Y1068, #2236), EGFR (#2232), pErk1/2 (#9101), Erk1/2 (#9102), pAKT (T308, #9257; S473, #9271), AKT (#2920), pSTAT3 (#9138), STAT3 (#9139), pFAK (#8556) and FAK (#13009), all from Cell Signaling Technologies; BMX (C-17, sc-8874) from Santa Cruz and ß-actin (A1978) from Sigma.

### Statistical analysis

The statistical analysis was conducted in SPSS 16.0 (SPSS Inc, Chicago, IL, USA). Pearson's chi-squared test was used on categorical variables. Two group comparisons were analyzed by the two-tailed Student's t test. A *p* value less than 0.05 was considered statistically significant.

## SUPPLEMENTARY MATERIALS FIGURES



## Abbreviations

BMX: Bone marrow tyrosine kinase gene in chromosome X
BTK: Bruton's tyrosine kinase
FBS: Fetal bovine serum
MTT: 3-(4, 5-dimethylthiazol-2-yl)-2, 5-diphenyltetrazolium bromide
NSCLC: non-small cell lung cancer
