# Supplementary material for: A novel BMX variant promotes tumor cell growth and migration in lung adenocarcinoma
Source: Oncotarget. 2017 Apr 3;8(20):33405–15. doi: 10.18632/oncotarget.16796 (PMC5464877; doi:10.18632/oncotarget.16796)
Supplement: Supplementary file 1 [file oncotarget-08-33405-s001.pdf]

A novel BMX variant promotes tumor cell growth and migration in lung adenocarcinoma

Supplementary Materials

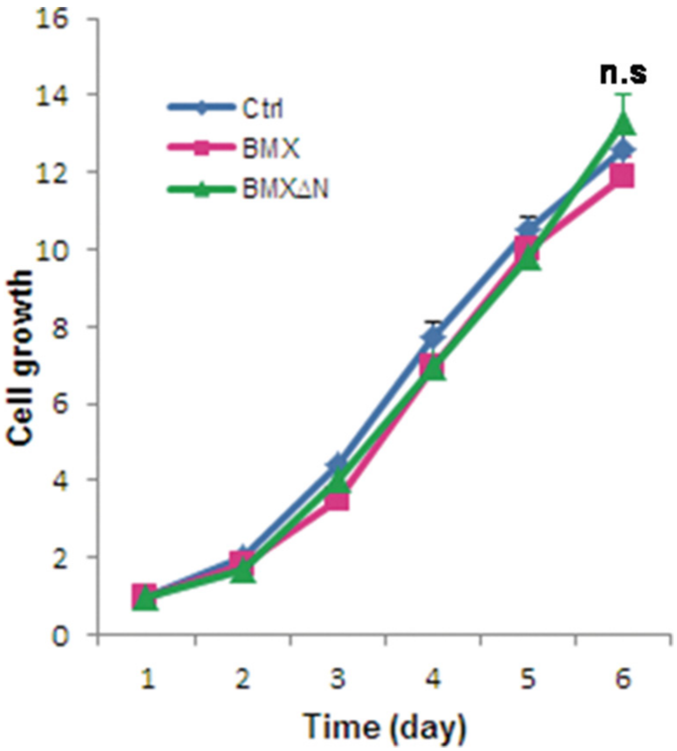

Supplementary Figure 1: Cell proliferation detection of PC9 cells with or without BMX and BMXΔN expression. Statistical analysis was performed using Student's *t* test (n.s: not significant).
